# A novel modified RANKL variant can prevent osteoporosis by acting as a vaccine and an inhibitor

**DOI:** 10.1002/ctm2.368

**Published:** 2021-03-17

**Authors:** Young Jong Ko, Hong Moon Sohn, Yuria Jang, Mineon Park, Bora Kim, Beomchang Kim, Jae‐Il Park, Hoon Hyun, Byeongseok Jeong, Chansik Hong, Wonbong Lim

**Affiliations:** ^1^ Laboratory of Orthopaedic Research Chosun University Hospital Dong‐Gu Gwangju Republic of Korea; ^2^ Department of Orthopaedic Surgery Chosun University Hospital Dong‐Gu Gwangju Republic of Korea; ^3^ Korea Basic Science Institute Gwangju Center at Chonnam National University Gwangju Republic of Korea; ^4^ Department of Biomedical Sciences Chonnam National University Medical School Gwangju Republic of Korea; ^5^ Department of Physiology School of Medicine Chosun University Gwangju Republic of Korea; ^6^ Department of Premedical Science College of Medicine Chosun University Dong‐Gu Gwangju Republic of Korea

**Keywords:** immunotherapy, LGR4, osteoclastogenesis, RANK

## Abstract

**Background:**

The discovery of receptor activator of nuclear factor‐ĸB ligand (RANKL) as the final effector in the pathogenesis of osteoporosis has led to a better understanding of bone remodeling. When RANKL binds to its receptor (RANK), osteoclastic differentiation and activation are initiated. Herein, we propose a strategy using a novel RANKL variant as a competitive inhibitor for RANKL. The RANKL variant activates LGR4 signaling, which competitively regulates RANK and acts as an immunogen that induces anti‐RANKL antibody production.

**Methods:**

We modified the RANK‐binding site on RANKL using minimal amino acid changes in the RANKL complex and its counterpart receptor RANK and tried to evaluate the inhibitory effects on osteoclastogenesis.

**Results:**

The novel RANKL variant did not bind RANK in osteoclast progenitor cells, but activated LGR4 through the GSK3‐β signaling pathway, thereby suppressing activated T cell cytoplasmic nuclear factor calcineurin‐dependent 1 (NFATc1) expression and activity during osteoclastogenesis. Our RANKL variant generated high levels of RANKL‐specific antibodies, blocked osteoclastogenesis, and inhibited osteoporosis in ovariectomized mouse models. Generated anti‐RANKL antibodies showed a high inhibitory effect on osteoclastogenesis *in vivo* and *in vitro*.

**Conclusions:**

We observed that the novel RANKL indeed blocks RANKL via LGR4 signaling and generates anti‐RANKL antibodies, demonstrating an innovative strategy in the development of general immunotherapy.

## Graphical abstract

Schematic diagram of mRANKL‐MT3 in dual inhibitory effect against RANKL during osteoclastogenesis. The first effect of mRANKL‐MT3 is induced to RANKL‐LGR4 modulation of the RANKL–NFATc1 signaling cascade by a negative‐feedback mechanism, which controls osteoclast activity. The second effect is induced to anti‐RANKL generation by mutant RANKL, which inhibits osteoclastogenesis and bone erosion.

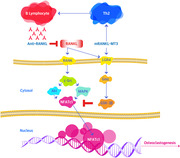

**Abbreviations**
ALD: alendronate
BMD: Bone Mineral Density
BMMs: bone marrow‐derived monocytes
BV/TV: bone volume over total volume
CT: computerized tomography
CTX‐1: cross‐linked C‐telopeptide of type 1 collagen
DC‐STAMP: dendritic cell‐specific transmembrane protein
ECD: extracellular domain
FSHR: follicle‐stimulating hormone receptor
GPR48: G‐protein coupled receptor 48
IgG: immunoglobulin G
LGR4: leucine‐rich repeat‐containing G protein‐coupled receptor 4
M‐CSF: macrophage colony‐stimulating factor
mRANKL: mouse RANKL
NFATc1: nuclear factor of activated T cells c1
OPG: osteoprotogerin
OSCAR: osteoclast‐associated receptor
OVX: ovariectomized
RANKL: receptor activator of NF‐kB ligand
Tb. N.: trabecular number
Tb. Th.: trabecular thickness
TRAP: tartrate‐resistant acid phosphate
TSHR: thyroid‐stimulating hormone receptor
VLPs: virus‐like particles
VOI: volume of interest

## 1 BACKGROUND

Osteoclasts are multinucleated cells that reabsorb bone tissue and play a pivotal role in the maintenance of bone homeostasis.^1^, ^2^ Aberrant differentiation or osteoclast function can lead to serious skeletal disorders such as osteoporosis and osteopetrosis.^3^ Therefore, fixing any underlying unbalanced osteoclast metabolism is highly important in bone disease treatment. Osteoclasts differentiate from hematopoietic stem cells upon stimulation with two key cytokines: the receptor activator of NF‐κB ligand (RANKL; also known as TNFSF11) and macrophage colony‐stimulating factor (M‐CSF).^4^, ^5^ RANKL is a key positive osteoclast regulator and several strategies to treat bone disease target RANKL, including cell‐specific RANKL deletion or treatment with anti‐RANKL monoclonal antibodies such as denosumab, which efficiently inhibit osteoclast activity and protect against severe bone erosion.^6^, ^7^ However, high costs and poor patient compliance are obstacles in the widespread application of these treatments in osteoporosis patients.^8^ Therefore, there is a special need to explore novel, efficient, and cost‐efficient methods to treat and prevent osteoporosis.

RANKL binding to the receptor activator of NF‐κB (RANK) receptor (also known as TNFRSF11A) drives osteoclast differentiation via this crucial signaling pathway.^9^, ^10^, ^11^ Membrane‐bound RANKL activates RANK to generate osteoclasts through cell‐to‐cell contact.^12^ The RANKL TNF‐like domain has been linked to manufactured virus‐like particles (VLPs) and introduced as RANKL vaccines that might be able to induce RANKL‐specific antibodies, ameliorate RANKL activity, and prevent bone loss in an osteoporotic mouse model.^13^, ^14^ However, a vaccine containing VLP complexes may not only reduce natural tolerance but also stimulate osteoclast differentiation and immune complex deposition, potentially inducing pathological lesions.^15^ Because of these potential problems, VLP vaccine trials have not been conducted in clinical settings. Nevertheless, several studies showed that incorporating similar amino acids via point mutations of an original protein can help overcome autologous immune tolerance.^16^, ^17^ Recombinant proteins incorporating similar residues in their binding site that do not allow ligand binding can enhance immunogenicity, prevent immune tolerance, and produce numerous polyclonal antibodies. Previously, we generated a mutant RANKL vaccine by replacing a RANKL‐RANK binding site in murine RANKL with similar amino acids to induce a high level of anti‐RANKL antibodies and prevent OVX‐induced osteoporosis in mice.^16^

As another RANKL receptor, LGR4 could act as a target receptor because it has a similar binding structure with RANK. LGR4, also known as GPR48, was recently described as LGR4‐ECD, which can antagonize excessive RANKL and play a role in the negative‐feedback mechanism that limits osteoclast function, especially in pathologic conditions.^18^, ^19^, ^20^ Further, LGR4 regulates multiple developmental signals through either classical G‐protein pathways or the potentiation of Wnt signaling.^21^ LGR4 is part of the LGR family, which includes another two members, follicle‐stimulating hormone receptor and thyroid‐stimulating hormone receptor, which regulate osteoclast differentiation and activity.^22^

We hypothesized that a similar structure between mutant and native RANKL compensates for RANKL‐RANK signaling, and the variant acts as another RANK receptor that negatively regulates osteoclast differentiation and bone resorption. In principle, native RANKL sequences could be used as an immunogen to treat osteoporosis, but this approach could transiently increase bone resorption via RANK activation. Thus, we evaluated novel RANKL variants to assess the potential use of modified RANKL as a vaccine. We also investigated whether LGR4 is another RANKL receptor. Next, we created a modified RANKL sequence that does not bind and activate RANK, but instead activates LGR4, which acts as a mild inhibitor of osteoclastogenesis. In this approach, the immunogen itself will not stimulate osteoclast activity, but will induce an immune response associated with the cross‐reaction with native RANKL, thereby blocking native RANKL activity and effectively immunizing the patient against their own RANKL.

## 2 METHODS

### 2.1 Site‐directed mutagenesis of mouse RANKL (mRANKL)

Amplification and cloning of the RANKL fragment and mutant RANKL candidates were carried out as mentioned in our previous study.^16^ The polymerase chain reaction (PCR) product was cloned into the NdeI/XhoI site in the pGEX‐4T‐1 vector (Promega, Madison, WI, USA) and mutations at sites 180, 189–190, and 223–224 were introduced using megaprimers (Table 1). The PCR product was transformed into *Escherichia coli* BL21‐Gold competent cells (Agilent, Santa Clara, CA, USA) by electroporation (5 ms, 12.5 kV/cm). The transformed *E. coli* cells were cultivated in Luria–Bertani (LB) broth with ampicillin (50 μg/mL, T&I, Daejeon, Korea). The cloned product was confirmed by a commercial sequencing service (SolGent Co., Daejeon, Korea). All sequence data were analyzed using Vector NTI Advance 9.1.0 (Invitrogen, Carlsbad, CA, USA).

**TABLE 1:** Megaprimers for site‐directed mutagenesis of mouse receptor activator of NF‐kB ligand (mRANKL)

Primer	Sequence
mRANKL‐NdeI mRANKL‐XhoI mRANKL(K180R)‐F mRANKL(K180R)‐R mRANKL(D189I, R190K)‐F mRANKL(D189I, R190K)‐R mRANKL‐MT1 (H223F, H224F)‐F mRANKL‐MT1 (H223F, H224F)‐R mRANKL‐MT2 (H223Y, H224Y)‐F mRANKL‐MT2 (H223Y, H224Y)‐R mRANKL‐MT3 (H223F, H224Y)‐F mRANKL‐MT3 (H223F, H224Y)‐R mRANKL‐MT4 (H223Y, H224F)‐F mRANKL‐MT4 (H223Y, H224F)‐R	5′‐CATATGAAGCCTGAGGCCCAGCCATTTGC‐3′ 5′‐CTCGAGGTCTATGTCCTGAACTTTGAAAGCC‐3′ 5′‐CCCATCGGGTTCCCATCGAGTCACTCTGTCCTCTTG‐3′ 5′‐CAAGAGGACAGAGTGACTCGATGGGAACCCGATGGG‐3′ 5′‐CTCTTGGTACCACATCAAGGGCTGGGCCAAGAT‐3′ 5′‐ATCTTGGCCCAGCCCTTGATGTGGTACCAAGAG‐3′ 5′‐AACATTTGCTTTCGGTTTTTTGAAACATCGGGAAGCG‐3′ 5′‐CGCTTCCCGATGTTTCAAAAAACCGAAAGCAAATGTT‐3′ 5′‐AACATTTGCTTTCGGTATTATGAAACATCGGGAAGCG‐3′ 5′‐CGCTTCCCGATGTTTCATAATACCGAAAGCAAATGTT‐3′ 5′‐AACATTTGCTTTCGGTTTTATGAAACATCGGGAAGCG‐3′ 5′‐CGCTTCCCGATGTTTCATAAAACCGAAAGCAAATGTT‐3′ 5′‐AACATTTGCTTTCGGTATTTTGAAACATCGGGAAGCG‐3′ 5′‐CGCTTCCCGATGTTTCAAAATACCGAAAGCAAATGTT‐3′

### 2.2 mRANKL purification

The recombinant plasmid carrying each RANKL variant was expressed using previously described methods.^16^ To obtain the bacterial lysate for affinity column chromatography, the cell pellet was resuspended in lysis buffer (phosphate‐buffered saline [PBS], 0.1% Tween 20, 1 mM ethylenediaminetetraacetic acid (EDTA) [pH 8.0], and 20 μM phenylmethylsulfonyl fluoride [PMSF]) and homogenized by sonication (Vibra Cell VCX500; Sonics & Materials, Inc., Newtown, CT, USA) on ice. After sonication, recombinant GST‐tagged RANKL was purified using a Glutathione Sepharose 4 M resin column (GE Healthcare, Uppsala, Sweden). For endotoxin removal, High‐Capacity Endotoxin removal resin (Pierce Biotechnology Inc., Rockford, IL, USA) was used. The protein samples were separated by sodium dodecyl sulfate polyacrylamide gel electrophoresis (SDS‐PAGE). After separation, the SDS gel was stained with Coomassie brilliant blue G‐250 and images were acquired with a digital scanner (EPSON, USA).

### 2.3 Osteoclast differentiation from primary cultured BMMs and TRAP staining

Bone marrow cells were flushed from the femurs of 6‐week‐old female C57BL/6 mice, cultured in alpha‐MEM containing 10% fetal bovine serum (FBS; Thermo Fisher Scientific Inc. Waltham, MA, USA) and 30 ng/mL M‐CSF (R&D systems, Minneapolis, MN, USA) after treatment with RBC lysis buffer (Gibco, Gaithersburg, MD, USA). After incubation for 3 days, supernatant cells were removed. Adherent cells were used for BMMs. Then, osteoclast differentiation was induced by adding 30 ng/mL M‐CSF and 75 ng/mL sRANKL (R&D Systems) or isolated RANKL for 4 days. Multinucleated cells were stained using a TRAP assay kit (KAMIYA BIOMEDICAL Co., Seattle, WA, USA), and TRAP‐positive cells with more than five nuclei were identified as osteoclasts.

### 2.4 Bone resorption assay

To observe bone resorption *in vitro*, BMMs were cultured in Corning Osteo Assay Surface 96‐well Multiple Well Plates (Sigma) with 30 ng/mL M‐CSF and 75 ng/mL sRANKL (R&D Systems) or isolated RANKL for 6 days. Then, the plates were washed with pure water.

### 2.5 F‐actin ring formation assay

To monitor actin ring formation, BMMs were grown on glass slides. After culture, the cells were fixed with 4% formalin, permeabilized with 0.5% Triton X100 in PBS for 5 min at room temperature, and incubated with 0.5 mg/mL TRITC‐labeled phalloidin for 30 min. The cells were then rinsed with PBS. F‐actin rings were visualized using a fluorescence ECLIPSE Ts2R microscope (Nikon, Tokyo, Japan).

### 2.6 Real‐time PCR

BMM cells were incubated with 30 ng/mL M‐CSF and 75 ng/mL sRANKL (R&D Systems) or isolated RANKL in 6‐well plates. Total RNA was extracted using TRIzol reagent (Invitrogen, Thermo Fisher Scientific, Inc.). The cDNA was synthesized from 2 μg total RNA using ReverTra Ace qPCR RT Master Mix (TOYOBO, Osaka, Japan). Real‐time PCR was conducted on a CFX Connect Real‐Time PCR Detection System (Bio‐Rad, Hercules, CA, USA) in a reaction mixture (total volume, 20 μL) containing IQ SYBR Green Supermix (Bio‐Rad), 10 pmol forward primer, 10 pmol reverse primer, and 1 μg cDNA. The primer sequences used to target various genes are listed in Table 2.

**TABLE 2:** Primer sequence for real‐time polymerase chain reaction (PCR) in related with osteoclastogenesis

Primer	Sequence	Size(bp)
TRAP‐F	TAC CGT TGT GGA CAT GAC C	150
TRAP‐R	CAG ATC CAT AGT GAA ACC GC	
OSCAR‐F	CTG CTG GTA ACG GAT CAG CTC CCC AGA	310
OSCAR‐R	CCA AGG AGC CAG AAC CTT CGA AAC T	
NFATc1‐F	CAA CGC CCT GAC CAC CGA TAG	392
NFATc1‐R	GGC TGC CTT CCG TCT CAT AGT	
Atp6v0d2‐F	GAA GCT GTC AAC ATT GCA GA	191
Atp6v0d2‐R	TCA CCG TGA TCC TTG CAG AAT	
c‐fms‐F	GCG ATG TGT GAGCAA TG CAG T	341
c‐fms‐R	GAG CCG TTT TGC GTA AGA CCT G	
ATP6v0a3‐F	CGC CAC AGA AGA AAC ACT CA	247
ATP6v0a3‐R	CCC AGA GAC GCA AGT AGG AG	
c‐fos‐F	ATG GGC TCT CCT GTC AAC AC	336
c‐fos‐R	GGC TGC CAA AAT AAA CTC CA	
MMP‐9‐F	TCC AGT ACC AAG ACA AAG	183
MMP‐9‐R	TTG CAC TGC ACG GTT GAA	
Cathepsin K‐F	TGT ATA ACG CCA CGG CAA A	195
Cathepsin K‐R	GGT TCA CAT TAT CAC GGT CAC A	
DC‐STAMP(m)‐F	TGG AAG TTC ACT TGA AAC TAC GTG	322
DC‐STAMP(m)‐R	CTC GGT TTC CCG TCA GCC TCT CTC	
Calcitonin receptor‐F	ACC GAC GAG CAA CGC CTA CGC	272
Calcitonin receptor‐R	GCC TTC CAC GCC TTC AGG TAC	
Integrin β3‐F	TGA CTC GGA CTG GAC TGG CTA	414
Integrin β3‐R	CAC TCA GGC TCT TCC ACC ACA	
RANK‐F	CCA GGG GAC AAC GGA ATC A	492
RANK‐R	GGC CGG TCC GTG TAC TCA TC	
LGR4‐F	TAGGATTCAC TGGGACCCTA GTGCT	160
LGR4‐R	CAGTTTGTGA AGATGAGCCA AGA	
β‐actin‐F	GTC CCT CAC CCT CCC AAA AG	266
β‐actin‐R	GCT GCC TCA ACA CCT CAA CCC	

### 2.7 Co‐immunoprecipitation assay

BMM cells were incubated with 500 ng/mL mRANKL‐WT or mRANKL‐MT3 at 37°C for 45 min. Then, the cells were lysed in lysis buffer (20 mM Tris‐HCl pH 7.6–8.0, 100 mM NaCl, 300 mM sucrose, and 3 mM MgCl_2_ [buffer A] and 20 mM Tris pH 8.0, 100 mM NaCl, and 2 mM EDTA [buffer B]). Whole‐cell lysates were obtained by centrifugation and incubated with antibodies specific for RANK (Cell Signaling Technology, #14373S) and LGR4 (MyBioSource, #MBS468030) (dilution 1:100) and protein A Sepharose beads (Amersham Biosciences) for 2 h at room temperature. The immune complexes were washed three times using Tris‐buffered saline‐Tween buffer (TBST; 2.42 g/L, Tris‐HCl; 8 g/L, 0.1% Tween 20, pH 7.6) and examined by western blotting.

### 2.8 Flow cytometry analysis

BMM cells were incubated with 500 ng/mL mRANKL‐WT or mRANKL‐MT3 at 37°C for 45 min, and then were fixed with 4% paraformaldehyde. After washing, the cells were incubated with Alexa Fluor 647‐conjugated GST (26H1) anti‐mouse IgG (Cell Signaling Technology, 3368, 1:100), and PE‐conjugated CD14 (61D3) anti‐mouse IgG (Cell Signaling, Technology, 59896, 1:40) at 4°C for 1 h. The cells were washed and analyzed by fluorescence‐activated cell sorting (FACS) (BECKMAN COULTER, Indianapolis, IN, USA). The flow cytometry data were analyzed using Kaluza Analysis Software (BECKMAN COULTER).

### 2.9 Western blot analysis

BMM cells were incubated with 1000 ng/mL sRANKL (R&D Systems) or isolated RANKL in 6‐well plates. At the indicated time, the cells were washed with PBS (pH 7.4). Then, the cells were lysed at 4°C in lysis buffer (50 mM Tris‐HCl, pH 7.5, 1% NP‐40, 150 mM NaCl, 0.02% sodium azide, 1 mg/mL pepstatin A, 2 mg/mL aprotinin, 20 mg/mL leupeptin, and 150 mg/mL PMSF). Approximately 30 mg of cell lysate was separated by 10% SDS‐PAGE and transferred to a polyvinylidene difluoride membrane (Amersham, Piscataway, NJ, USA). The membrane was blocked for 30 min with 5% albumin in TBST and rinsed with TBST. The membrane was incubated for 3 h at 4°C with the following primary antibodies: AKT (Cell Signaling Technology, 1:1000), phospho‐AKT (Cell Signaling Technology, #9271S, 1:1000), p38 (Cell Signaling Technology, 9212S, 1:1000), phospho‐p38 (Cell Signaling Technology, #9211S, 1:1000), ERK (Cell Signaling Technology, 9102S, 1:1000), phospho‐ERK (Cell Signaling Technology, #9101S, 1:1000), JNK (Cell Signaling Technology, 9252S, 1:1000), phospho‐JNK (Cell Signaling Technology, #9251S, 1:1000), GSK‐3β (Cell Signaling Technology, 9315S, 1:1000), phospho‐GSK‐3β (Cell Signaling Technology, #9336S, 1:1000), Src (Cell Signaling Technology, 2108S, 1:1000), phospho‐Src (Cell Signaling Technology, #2105S, 1:1000), NF‐κB p65 (Cell Signaling Technology, #3034, 1:1000), phospho‐p65 (Cell Signaling Technology, #3031, 1:1000), RANK (Cell Signaling Technology, 4845S, 1:1000), G_αq_ (Cell Signaling Technology, 14373S, 1:1000), LGR4 (MyBioSource, MBS468030, 1:500), 20112360C‐1, and GAPDH (Santa‐Cruz Biotechnology, 1:2500). After rinsing with TBST, the membrane was incubated for 1 h with anti‐rabbit or anti‐mouse horseradish peroxidase‐conjugated secondary antibody (1:2000; Santa Cruz Biotechnology). Finally, the membrane was washed in TBST, and protein immunoreactivity was detected using an enhanced chemiluminescence detection kit (Sigma Aldrich Inc., Saint Louis, MO, USA).

### 2.10 Intracellular Ca^2+^ measurements with Fura‐2

Ratiometric [Ca^2+^] measurements were carried out using Fura‐2‐AM (Molecular Probes, USA). Cells were suspended and incubated with 2 μM Fura‐2‐AM for 30 min. Fluorescence was measured at 340/380 nm dual excitation and 510 nm emission using a pE‐340 Fura illuminator (CoolLED, UK). Images were acquired on an electron‐multiplying 22 MHz (16 bit) CCD camera (C9100‐23B, Hamamatsu, Japan) with NIS‐Elements imaging software (Nikon, Japan). Each image was exposed for 5 s. The basal 340/380 nm fluorescence signal of the cells in the field was monitored for 3 min, and then the cells were treated with 250 ng/mL RANKL at 37°C. The experiments were performed in a solution containing (in mM) 135 NaCl, 5 KCl, 10 HEPES, 2 CaCl_2_, 1 MgCl_2_, and 10 glucose with the pH adjusted to 7.4 using NaOH. Extracellular Ca^2+^‐free (0 CaCl_2_) solution was used for RANKL‐induced Ca^2+^ release from the ER.

### 2.11 Nuclear extract preparations

Cells were rinsed with PBS, and Solution A (10 mM HEPES, 1.5 mM MgCl2, 10 mM KCl, 0.5 mM DTT, and 0.05% NP40 pH 7.9, 0.5 mL) was added to the cells. Then, the cells were centrifuged at 805 × *g* for 10 min at 4°C. The cytoplasmic constituents were contained in collected supernatants. To acquire nuclear extracts, 0.4 mL solution B (300 mM NaCl, 0.2 mM EDTA, 5 mM HEPES, 0.5 mM DTT, 1.5 mM MgCl_2_, and 26% glycerol (v/v), pH 7.9) was added and mixed thoroughly. Then, the mixed solutions were centrifuged at 12 000 × *g* for 3 min. The supernatants contained mostly nuclear proteins. The proteins in both nuclear and cytosol extracts were quantified using a BCA protein assay kit (Thermo Scientific, IL) and analyzed by Western blot.

### 2.12 Immunofluorescence analysis

BMM cells were incubated with 1000 ng/mL isolated RANKL in four‐well chamber slide. After 1 h, the cells were fixed in 4% paraformaldehyde. Then, the cells were incubated with rabbit anti‐NFATc1 (1:200, diluted in PBST containing 5% BSA) for 2 h. After washing with PBST, the cells were incubated with FITC‐labeled goat anti‐rabbit IgG antibody (10 μg/mL diluted in PBST containing 5% BSA) for 1 h in the dark and washed with PBST for 10 min. Immunolabeled cells were counter‐stained with 4′,6′‑diamidino‑2‑phenylindole (DAPI) contained in Pro‐Long Gold mounting solution (Invitrogen; Thermo Fisher Scientific, Inc., Waltham, MA, USA). Digital images were acquired at the Korea Basic Science Institute Gwangju Center using a TCS SP5 AOBS (Leica Microsystems, Heidelberg, Germany) laser‑scanning microscope.

### 2.13 Mice

Five‐week‐old female mice (BL‐6; Orient Bio Co. LTD, Seoul, South Korea) were housed under controlled 12hr light/dark cycle conditions and fed ad libitum. All animal experiments were carried out in compliance with institutional and governmental requirements approved by the Institutional Animal Care and Use Committee (CIACUC2018‐S0012‐1) of Chosun University, Gwangju, South Korea.

### 2.14 Mice immunization and ovariectomy

Fifty mice were equally divided into five groups. Group 1 (Sham group) was intraperitoneally injected with PBS; Group 2 (OVX group) was ovariectomized and injected with PBS vehicle; Group 3 (IM) was immunized with mutant RANKL without OVX; Group 4 (OVX+ALD) was ovariectomized and injected with sodium alendronate (0.1 mg/kg); and Group 5 (OVX+IM) was ovariectomized and immunized with mutant RANKL. The protein used for immunization was mixed with aluminum hydroxide as an adjuvant. The interval for each immunization was 2 weeks and the amount of protein used for immunization was 100 μg/kg per mouse according to the indicated schedule. Mouse ovariectomies were performed as described by Sophocleous et al.^23^ Surgical procedures in the sham and IM group were the same as those used in the OVX group, except the ovaries were left intact. After recovering from surgery for 12 weeks, all animals were fasted for 24 h and sacrificed. Finally, femur bone and blood samples were collected for further study.

### 2.15 Micro‐CT imaging data acquisition

Micro‐CT scanning of the distal femur was distally initiated at the level of growth plate using a Quantum GX (PerkinElmer, Hopkinton, MA, USA) micro‐CT imaging system located at the Korea Basic Science Institute in Gwangju, Korea. The scanning X‐ray source was set to 88 mA and 90 kV, with a 10 mm field of view (scanning time, 4 min; voxel size, 20 μm). The 3D architecture images were acquired using 3D Viewer commercial software included with the Quantum GX system. The 3D images were obtained at 4.5 μm resolution. After scanning, the bone structure parameters were analyzed with Analyze 12.0 software (AnalyzeDirect, Overland Park, KS, USA) using the ROI tool. Femur bone mineral density was estimated using hydroxyapatite (HA) Phantom (QRM‐MicroCT‐HA, Quality Assurance in Radiology and Medicine GmbH, Germany) scanning using the same parameters. Parameter values are shown as mean ± standard deviation (SD).

### 2.16 Detection of anti‐RANKL antibody

Sera were obtained 12 weeks after the third immunization. The specific anti‐serum titers against sRANKL were analyzed by enzyme‐linked immunosorbent assay (ELISA). Briefly, 5 mg/mL sRANKL protein (R&D Systems) was coated on the bottom of MaxiSorp microtiter plates (Thermo Fisher Scientific Inc.). Mice sera were diluted in PBS with 1% BSA and incubated in the pre‐coated plates for 2 h. After washing with PBS, bound IgG was evaluated using horseradish peroxidase‐conjugated goat anti‐mouse IgG antibody (1:10,000, ProteinTech Group, Chicago, USA). Absorbance at 450 nm was measured using a microplate reader (BioTek, Winooski, VT, USA).

### 2.17 Analysis of serum sRANKL, OPG, and CTX

The amount of sRANKL, OPG, and CTX in mice sera were measured using a commercial ELISA kit (R&D Systems, MN, USA). Sample absorbance was measured at 450 nm on a microplate reader (BioTek). This reading was then subtracted from absorbance recorded at 570 nm.

### 2.18 Histological analysis of mouse tissues

Femurs were dissected, immersed in 4% formaldehyde, and decalcified in 7% EDTA with 0.5% paraformaldehyde for 40 days before processing. To analyze longitudinal sections of distal femurs, decalcified tissues were paraffin‐embedded and 2–3‐μm‐thick sections were cut, mounted on glass slides, and rehydrated using graded alcohol. The tissue sections were stained with hematoxylin/eosin (Shandon Varistain 24‐4, Histocom, Vienna, Austria), and images were acquired using an ECLIPSE Ts2R inverted microscope (Nikon).

### 2.19 TRAP staining of mouse tissues

To evaluate TRAP activity, tissue sections were deparaffinized, rinsed in PBS, and incubated with a solution containing 50 mM sodium acetate (pH 5.2), 0.15% Naphtol‐AS‐TR‐phosphate, 50 mM sodium tartrate, and 0.1% Fast Red T.R. (Sigma Aldrich Chemie Gmbh, Taufkirchen, Germany) for 30–40 min at room temperature. Then, the sections were rinsed in PBS and counterstained with 0.2% methyl green.

### 2.20 Immunohistochemical analysis of bone specimens

Paraffin sections were deparaffinized in three xylene washes and rehydrated in graded ethanol solutions. For antigen retrieval, the slides were placed in 0.01 M citrate buffer (pH 6.0) and heated in a steamer for 30 min. Endogenous peroxidases were quenched by incubating the samples with 3% hydrogen peroxide for 20 min at room temperature. The sections were incubated overnight at 4°C using 1:50 anti‐cathepsin K (Santa‑Cruz Biotechnology Inc.). Sections were then incubated for 30 min with biotinylated secondary antibody (LSAB system HRP kit; Dako Cytomation, Glostrup, Denmark), rinsed in PBS, and incubated for 30 min with a streptavidin‑peroxidase conjugate (LSAB; DakoCytomation). The reaction was developed for 5 min using 3,30‑diaminobenzidine tetrahydrochloride (Sigma‑Aldrich; Merck KGaA). The slides were counterstained in hematoxylin, dehydrated, and coverslipped. Negative and positive controls were simultaneously analyzed. The positive controls were mammary tissues. The slides were imaged using an inverted microscope (Nikon).

### 2.21 Measurement of mutant RANKL‐specific splenocyte responses

Mice were sacrificed by cardiac puncture in sham or immunized mice. The spleens were immediately removed, and mononuclear splenocytes were collected by density gradient centrifugation using Lymphoprep (Axis‐Shield Poc As, Oslo, Norway). Splenocytes (1×10^6^/mL) were suspended in RPMI 1640 media including 10% FBS. The cells were stimulated with 100 ng/mL sRANKL or mutant RANKL for 48 h. The cultured supernatants were collected and IL‐4, IL‐10, and IFN‐γ levels were determined by ELISA kits (R&D Systems).

### 2.22 Isolation of anti‐RANKL IgG from immunized mice

Mouse peripheral blood samples were prepared by centrifugation at 1,000 g for 15 min and diluted 1:15 with Protein G IgG binding buffer (Thermo Fisher Scientific Inc.). Diluted samples were loaded onto a Capturem Protein G Maxiprep Column (TaKaRa, Tokyo, Japan) and centrifuged at 2,000 g for 2 min. After washing the column with Protein G IgG binding buffer and PBS, the antibody was recovered using IgG Elution buffer (Thermo Fisher Scientific Inc.) and neutralized with pH neutralization buffer (1 M Tris, pH 9). Fractions containing the antibody were desalted using Slide‐A‐Lyzer MINI Dialysis device with a 10 kDa molecular weight cut‐off (Thermo Fisher Scientific Inc.). The purity of IgG preparations was analyzed by SDS‐PAGE.

### 2.23 Establishment of the mouse model of sRANKL‐induced osteoporosis

Thirty mice were equally divided into three groups. The sham group (Group 1) was intraperitoneally injected with PBS; the sRANKL + Control IgG group (Group 2) was given sRANKL with IgG from negative control mice, and sRANKL + anti‐RANKL (Group 3) was treated with sRANKL and anti‐RANKL IgG from immunized mice according to the indicated schedule (Figure 5A).

### 2.24 Statistical analysis

Fisher's exact tests were carried out when comparing two categorical data. Two continuous variables were tested using Wilcoxon rank‐sum tests, unless otherwise noted. Multiple testing corrections were carried out using the Benjamini–Hochberg procedure. All in vitro studies were conducted at least in triplicate. All quantitative results are presented as means ± SD. Statistical significance between the cell culture groups was determined using two‐tailed Student's t‐tests. Primary cell comparisons and all animal studies were analyzed with two‐way repeated measure ANOVA with Bonferroni multiple‐comparisons test. All reported *P*‐values were two‐sided, and *P* < 0.05 were considered statistically significant. All statistical analyses were carried out using GraphPad Prism Version 7 (GraphPad Software Inc.) and R software v3.2.3.

## 3 RESULTS

### 3.1 Selection of mRANKL variants for inhibition of osteoclastogenesis

**FIGURE 1:**
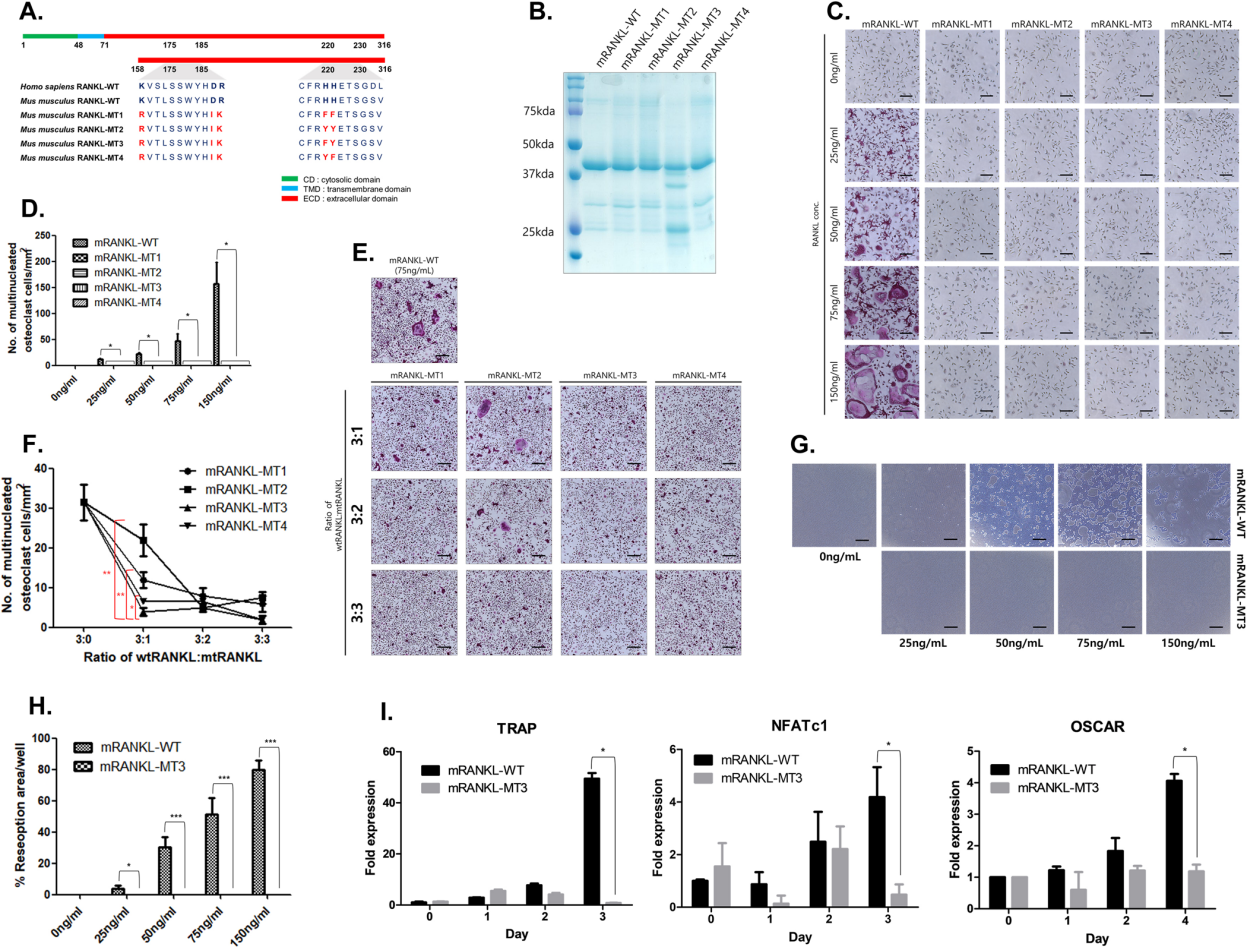
Selection of RANKL variants. A, Amino acid sequence of the RANKL protein in human, mouse, and mutant RANKL transformants. B, SDS‐PAGE of RANKL produced in *E. coli* after IPTG induction. C, Effects of RANKL variants on the generation of tartrate‐resistant acid phosphatase (TRAP)‐positive multinucleated cells. TRAP‐positive cells were visualized under a light microscope (100× magnification). The scale bar indicates 20 μm. D, Quantification of TRAP‐positive osteoclasts. E, Effect of RANKL‐MT treatment on the development of TRAP‐positive multinucleated cells in the presence of RANKL‐WT (75 ng/mL). F, Counted osteoclasts (TRAP‐positive cells). *P < .05 and **P < .01 when comparing RANKL‐MT3 with others, respectively. G, Effect of dose‐dependent RANKL‐MT3 treatment on bone resorption activity compared to RANKL‐WT (75 ng/mL). H, Resorption pits were quantified to investigate osteoclast activity. (i) TRAP, NFATc1, and OSCAR mRNA expression were analyzed by RT‐PCR. The data were normalized to GAPDH expression and are shown as the mean ratio ± SD from three separate experiments. *P < .05 and ***P < .001 when comparing RANKL‐WT with RANKL‐MT3

To find an optimal RANKL mutant that does not induce osteoclastogenesis, we selected four candidates with modified residues at the RANK binding site (Figure 1A). The RANKL‐RANK binding sites are K180, D189, R190, H223, and H224, which are conserved between humans and mice. We selected four mutant RANKLs to be purified. Their size corresponded to the wild‐type RANKL (39 kDa; Figure 1B). Tartrate‐resistant acid phosphate (TRAP) activity was absent for all RANKLs even at 150 ng/mL (Figure 1C and D).

To investigate the optimized inhibitory effect against wild‐type RANKL, bone marrow‐derived monocytes (BMMs) were treated with purified mutant and wild‐type RANKL (Figure 1E and F). Among them, mRANKL‐MT3 was the most effective at inhibiting TRAP activity, even at a 3:1 wild type:mutant ratio. Therefore, we used mRANKL‐MT3 for subsequent experiments.

We used bone‐resorption and F‐actin ring formation assays to investigate osteoclast activity. In mature osteoclasts treated with wild‐type RANKL, we observed numerous resorption pits and a ring of intracellular F‐actin filaments in the sealing zone (Figure 1 G, H; Figure S1A). However, with 150 ng/mL mRANKL‐MT3 treatment, no resorption pits or F‐actin rings were observed.

TRAP, NFATc1, and OSCAR, mRNA expression, which is associated with osteoclastogenesis, was down‐regulated in mRANKL‐MT3‐induced BMMs compared with expression in mRANKL‐WT induced BMMs (Figure 1I). Also, DC‐STAMP and cathepsin K mRNA, which are related to late osteoclastogenesis, showed expression patterns similar to OSCAR expression (Figure S1B).

### 3.2 Comparative inhibitory effects of mRANKL variants on wild type RANKL‐induced osteoclastogenesis

**FIGURE 2:**
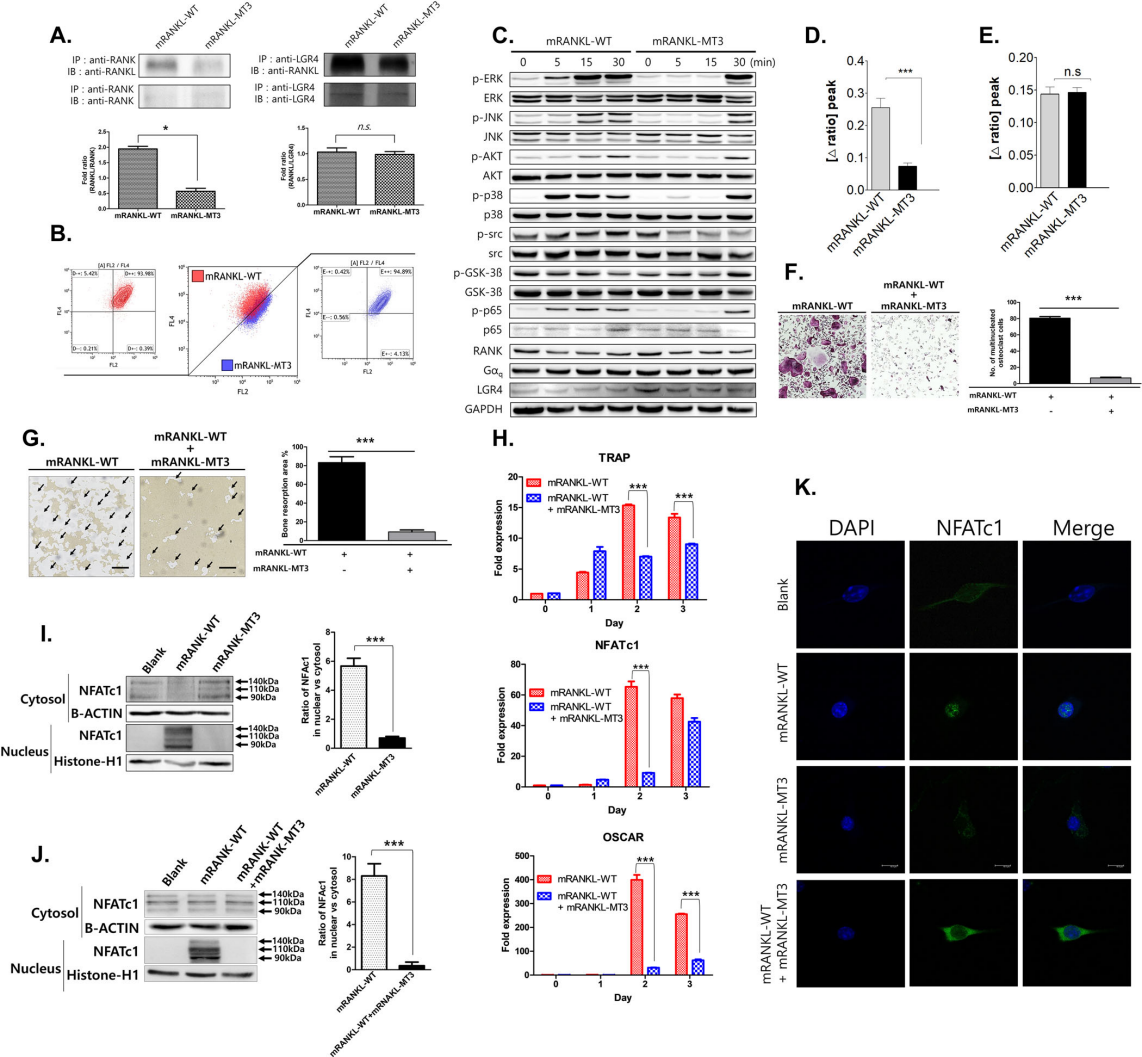
Comparative inhibition of osteoclastogenesis by RANKL variants. A, Co‐IP for RANK‐ or LGR4‐binding RANKL variants in BMMs. Each blot was obtained under the same experimental conditions. B, Representative flow cytometry plots showing GST‐RANKL‐bound BMMs. The *Y*‐axis represents GST binding by BMMs. CD14 is used as a monocyte marker. C, Western blots of RANK and LGR4 signaling pathway proteins. GAPDH was used as a loading control. The results are representative of three separate experiments with comparable results. D, Intracellular calcium concentration ([Ca^2+^]_i_) was measured in BMMs treated with 250 ng/mL mRANKL‐WT or 250 ng/mL mRANKL‐MT3. E, BMMs were preincubated in Ca^2+^‐free solution for 5 min to block plasma membrane Ca^2+^ influx. [Ca^2+^]_i_ was measured in calcium‐free solution. Data were expressed as mean ± SD. ***P < .001 and n.s., not significant. F, Effect of RANKL‐MT treatment on the generation of TRAP‐positive multinucleated cells in the presence of RANKL‐WT (150 ng/mL). G, Effect of RANKL‐MT treatment on bone resorption activity. The arrow indicates the resorptive area (H) TRAP, NFATc1, and OSCAR mRNA expression were analyzed by RT‐PCR. I, NFATc1 nuclear translocation was analyzed by Western blot analysis of cytosolic and nuclear fractions. Histone‐H1 from the nuclear fraction or β‐actin from the cytosol were used as loading controls. Densitometric analysis of NFATc1 in cytosolic and nuclear fractions represents the mean ratio ± SD of three separate experiments. ***P < .001 when comparing RANKL‐WT with RANKL‐MT3. n.s., not significant. J, NFATc1 nuclear translocation was analyzed by comparing mRANKL‐WT and mRANKL‐WT + mRANKL‐MT3. ***P < .001 when comparing RANKL‐WT with mRANKL‐WT + mRANKL‐MT3. K, NFATc1 nuclear translocation observed by confocal microscopy. Immunofluorescence images were acquired by staining for NFATc1 (green) and nuclei (blue). Magnification = 200×. The scale bar represents 20 μm

To investigate the interaction of mRANKL variants and RANK, the co‐immunoprecipitation (Co‐IP) assays were carried out using mRANKL‐WT/mRANKL‐MT3 and RANK/LGR4 (Figure 2A). The mRANKL‐WT strongly bound to both LGR4 and RANK, but mRANKL‐MT3 only bound LGR4. Further, GST‐tagged RANKL binding in BMMs was counted by flow cytometry to compare the interaction between BMMs and each RANKL variant (Figure 2B). The square of the D+‐region was 0.39% in the mRANKL‐WT group. The squared D+‐region increased to 4.13% in the mRANKL‐MT3 group. This implies a weaker interaction between BMMs and RANKL‐MT3 compared to wild‐type RANKL interactions, because the BMM and RANKL‐MT3 complex shifted the binding histogram to the left (Figure S2a). In addition, wild‐type RANKL induced MAPK, AKT, NF‐κB p65, and GSK‐3 phosphorylation, which is involved in LGR4 signaling (Figure 2C). Mutant mRANKL‐MT3 also induced GSK‐3 phosphorylation, but caused lower MAPK, AKT, and NF‐κB p65 phosphorylation levels. Furthermore, src phosphorylation, which is involved in RANK signaling, was significantly lower in mRANKL‐MT3‐induced BMMs than in mRANKL‐WT‐induced BMMs. These data suggest that mRANKL‐MT3 employs signaling transfer from LGR4 via src phosphorylation and without RANK signaling, in contrast to mRANKL‐WT. Because G_αq_ activation leads to intracellular calcium release, we subsequently used calcium imaging to examine whether mRANKL‐WT and mRANKL‐MT3 induce intracellular calcium release through LGR4. Calcium influx was significantly increased by mRANKL‐WT treatment compared to mRANKL‐MT3 treatment (Figure 2D). However, when an src inhibitor was used, calcium influx was similar after mRANKL‐WT and mRANKL‐MT3 treatment (Figure 2E). Therefore, our results indicate that both wild type and MT3 RANKL trigger LGR4‐mediated signaling via G_αq_ and GSK‐3.

TRAP activity was significantly inhibited by mRANKL‐MT3, while bone resorption was inhibited in the presence of mRANKL‐WT (Figure 2F and G). In addition, mRANKL‐MT3 led to a slight decrease in NFATc1 levels. TRAP, OSCAR, and other mRNA associated with osteoclastogenesis were significantly downregulated in a competitive inhibition assay using mRANKL‐WT (Figure 2H; Figure S2b). NFATc1 translocation into the nucleus was not detected in mRANKL‐MT3‐treated BMMs compared to that in mRANKL‐WT (Figure 2I). Even in the presence of mRANKL‐WT, mRANKL‐MT3 significantly inhibited NFATc1 translocation (Figure 2J). NFATc1 was present in mRANKL‐WT‐treated BMM nucleoli, but was not detected in the nucleus and cytosol of mRANKL‐MT3‐treated BMMs (Figure 2k, Supplementary Figure 2c). These results further support that mRANKL‐MT3 inhibits osteoclastogenesis stimulated by mRANKL‐WT and is an efficient RANKL inhibitor.

### 3.3 Effect of mRANKL‐MT3 in an ovariectomy‐induced osteoporosis model

**FIGURE 3:**
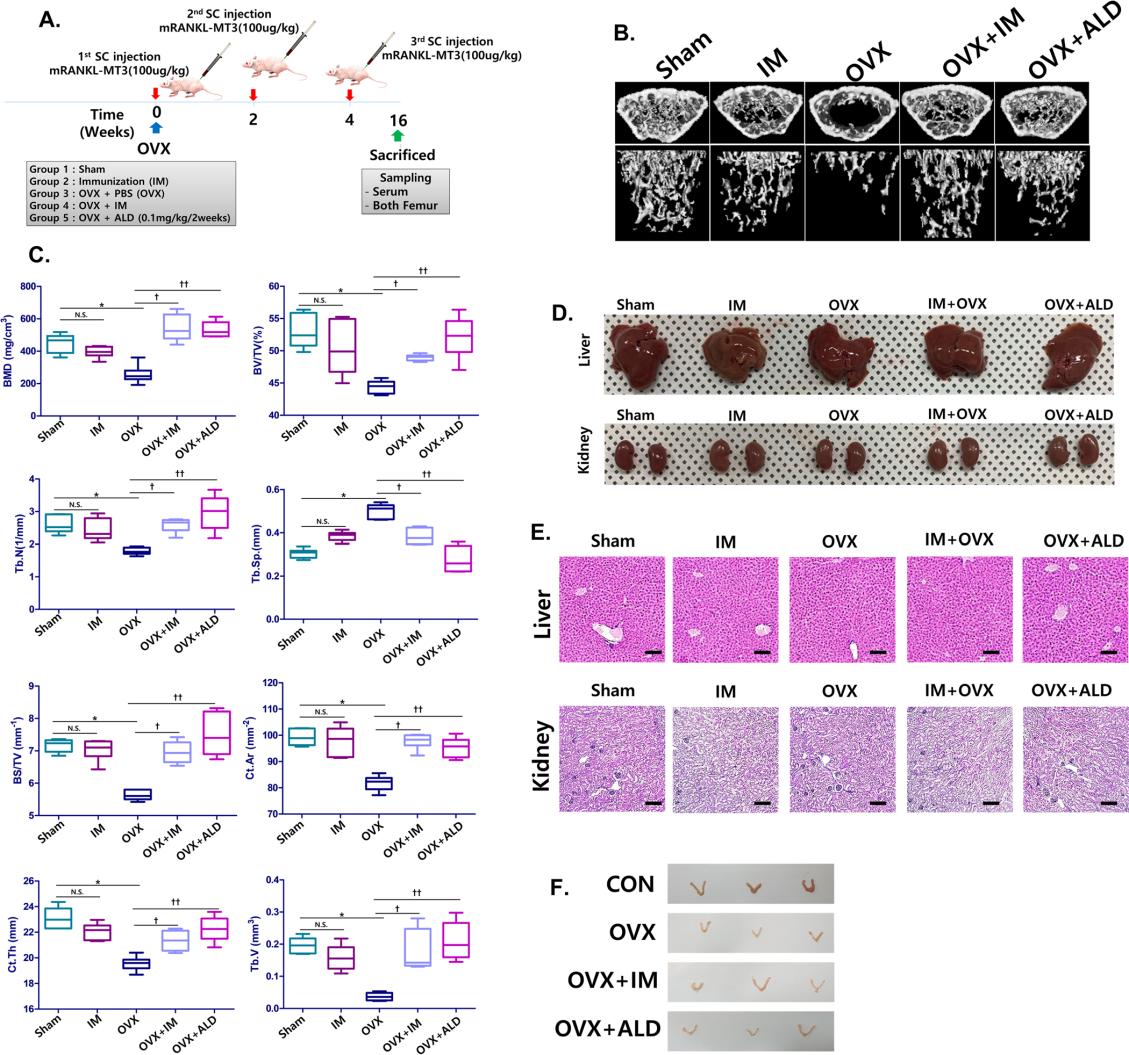
The effect of mRANKL‐MT3 immunization on ovariectomized mouse femurs. A, Immunization and sampling schedule in ovariectomized (OVX) mice. B, Three‐dimensional micro‐CT images revealed the trabecular bone architecture of the volume of interest in Sham, ovariectomized (OVX), mRANKL‐MT3‐immunized (IM), mRANKL‐MT3‐immunized ovariectomized (OVX+IM), mRANKL‐MT3 immunized, ovariectomized, and treated with alendronate (OVX+ALD) mouse femurs (n = 10 images taken in total, one image for each mouse). C, Bone mineral density (BMD), bone volume/trabecular volume (BV/TV), trabecular number (Tb. N.), trabecular separation (Tb. Sp.), bone surface density (Bone surface/total volume), Cortical Bone area (Ct. Ar.), cortical bone thickness (Ct. Th.) and trabecular volume (TV) are shown. Error bars are mean ± SD. *P < .05 for Sham versus OVX group, †*P* < .05 for OVX versus OVX+IM, ††*P* < .05 for OVX versus OVX+ALD and N.S.: non‐significant (*P* > .05). D, Extracted liver and kidney morphology between groups. E, Histomorphometric analysis images of liver and kidney. Magnifications are 100×. Size bar is 20 μm. F, Extract uterus morphology between groups

To demonstrate the therapeutic effect of RANKL immunization on bone resorption, ovariectomized (OVX) mice were immunized three times with mRANKL‐MT3 (Figure 3A). When we examined 3D images and the trabecular bone architecture of the distal femur, the trabecular bones from OVX mice immunized with mRANKL‐MT3 (OVX+IM) were significantly thicker and denser than those of OVX mice and alendronate‐treated OVX mice (OVX+ALD; Figure 3B). Also, BMD, BV/TV, Tb. N in the OVX+IM group were significantly increased compared to those in the OVX and OVX+AL groups (Figure 3C). Interestingly, the IM‐treated group showed no significant difference when compared to the sham‐OVX group (Sham). We examined liver and kidney morphology using visual observation and H&E staining, but there was no significant difference between the groups (Figure 3D and E). Nevertheless, the uteri in the OVX, OVX+ALD, and OVX+IM groups was thinner than that of controls, indicating OVX efficiency (Figure 3F).

**FIGURE 4:**
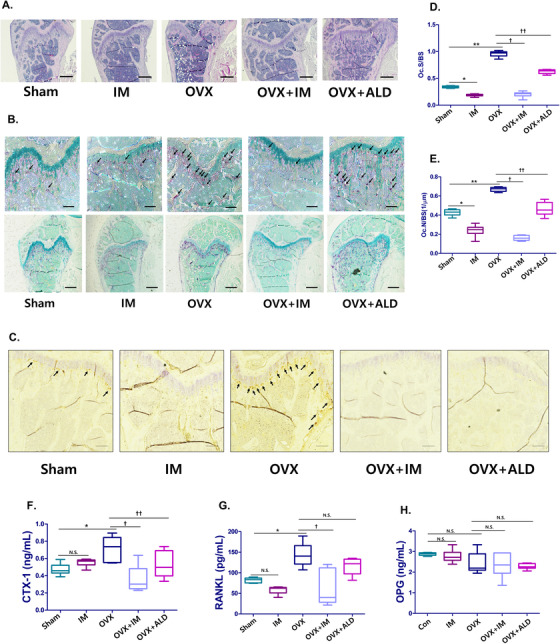
Histological examinations and image analysis on ovariectomized mouse femurs after immunization with mRANKL‐MT3. A, Histomorphometric analysis images. Magnifications are 20×. The scale bar represents 500 μm. B, TRAP staining images (n = 20 images taken in total, two images for each mouse) of femurs. The arrow indicates TRAP positive cell. Magnification is 100× at upper and 20× at lower. The scale bar represents 50 μm. C, Cathepsin K IHC images of femurs. Magnifications are 200×. Size bar is 10 μm. D, Parameters of femur osteoclasts. Oc.S/BS, osteoclast surface per bone surface. E, Oc.N/BS, osteoclast number per bone surface. F, CTX‐1 and (G) RANKL and (H) OPG levels in mouse sera. D‐H, Error bars are mean ± SD. *P < .05 for Sham versus OVX, **P < .05 for Sham versus IM, †*P* < .05 for OVX versus OVX+IM, ††*P* < .05 for OVX versus OVX+ALD and N.S., non‐significant (*P* > 0.05)

The next critical question was whether mRANKL‐MT3 immunization can prevent OVX‐induced bone loss. In a histological assay using H&E staining, we observed that the trabecular bone was thin, with a severely disrupted and fragmented network structure in OVX mice (Figure 4A). Some of the interstitial spaces between cells of trabecular bones expanded in size. However, the trabecular bone from mice femurs belonging to OVX+IM groups exhibited a normal arrangement pattern and a thick, dense network with minimal spaces, as in Sham and OVX+ALD mice. Next, we examined histological sections of femurs for TRAP and cathepsin K expression. In the TRAP staining results, OVX mice showed more TRAP‐positive osteoclasts compared to Sham mice, while OVX mice treated with mRANKL‐MT3 had fewer TRAP‐positive osteoclasts compared to OVX mice (Figure 4B). Cathepsin K immunostaining was similar to the TRAP staining results (Figure 4C). To quantify these data, the ratio of TRAP‐positive osteoclasts to trabecular bone surface (OCs/BS%) and the ratio of osteoclast number to bone area (OCs/mm^2^) were measured (Figure 4D and E). These results confirmed that the OCs/BS% and OCs/mm^2^ values in mRANKL‐MT3‐treated OVX mice were significantly smaller than those of OVX mice.

We investigated CTX‐1, soluble RANKL and osteoprotegerin (OPG) levels in mice serum to assess the effects of mRANKL‐MT3 on RANKL immunization status. Circulating CTX‐1 and soluble RANKL levels were higher in OVX mice (Figure 4F and G). However, mRANKL‐MT3 treatment significantly decreased circulating soluble RANKL and CTX‐1 levels. Further, OPG levels were unchanged in OVX mice with or without mRANKL‐MT3 (Figure 4H).

### 3.4 Antiserum titers for mice immunized with mRANKL‐MT3 and its effects as an mRANKL vaccine to treat osteoporosis *in vivo* and *in vitro*

**FIGURE 5:**
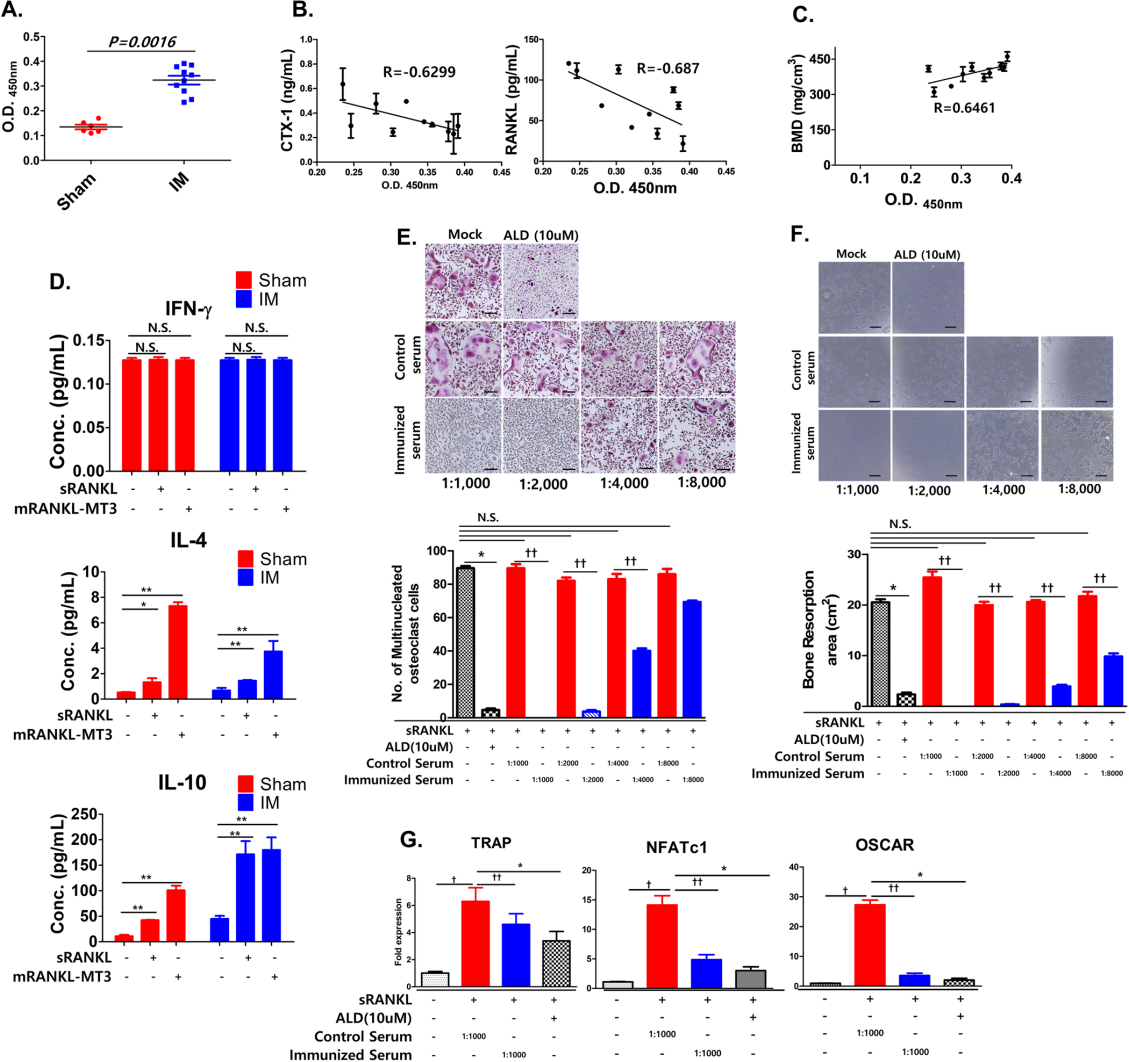
Antiserum titers after immunization with mRANKL variants and the effect on osteoclastogenesis. A, Serum titers for C57BL/6 mice immunized with or without mRANKL‐MT3‐substituted sRANKL proteins. Serum samples were obtained after 16 weeks. ELISAs were performed with commercial sRANKL. Before measurement, serum samples were diluted 1:1000 with 1% BSA in PBS buffer. B, Relationship with antiserum titer and CTX‐1 (left)/RANKL (right) in OVX‐IM mice. C, Relationship with antiserum titer and BMD in OVX‐IM mice. B, C, Pearson's correlation coefficient was measured between antiserum titer and (B) CTX‐1/RANKL or (C) BMD. All data are presented as the mean ± SD of three independent measurements. Statistical differences were determined by one sample *t*‐test. D, Effect of sRANKL or mRANKL‐MT3 induction on IFN‐γ, IL‐4, and IL‐10 expression in splenic lymphocytes cells from sham or immunized mice. All data are presented as the mean ± SD of three independent measurements. N.S., not significant (*P* > .05), *P < .05; **P < .01. E, Effects of dose‐dependent antiserum titer immunization on the generation of TRAP‐positive cells compared with control serum treatment. ALD treatment (10 μM) was used as a positive control. Representative TRAP staining (upper panel) and TRAP‐positive multinucleated cell quantification (lower panel) in the presence of sRANKL (75 ng/mL). F, Effect of dose‐dependent antiserum titer immunization on bone resorption compared with control serum treatment. E, F, Error bars are mean ± SD. *P < .05 for sRANKL versus sRANKL+ALD, ††*P* < .05 for sRANKL+Control Serum versus sRANKL+Immunized Serum and N.S., non‐significant (*P* > .05). G, TRAP, NFATc1, and OSCAR mRNA expression were analyzed by RT‐PCR for anti‐serum (1:1000)‐treated BMMs in the presence of sRANKL compared to ALD treatment. Error bars are mean ± SD. †*P* < .05 for Non versus sRANKL+Control Serum, ††*P* < .05 for sRANKL+ Control Serum versus sRANKL+Immunized Serum, and *P < .05 for sRANKL versus sRANKL+ALD

To determine whether vaccination with mRANKL‐MT3 induces anti‐mRANKL IgG antibodies, antibody titers against commercial mRANKL in the sham and the IM groups were determined by ELISA. As expected, mice immunized with mRANKL‐MT3 had markedly higher reactive serum mRANKL IgG titers, while Sham mice displayed insignificant serum IgG titers against mRANKL (Figure 5A).

To investigate the effect of anti‐mRANKL IgG antibodies on bone remodeling markers and soluble RANKL in the serum, relationships with anti‐RANKL IgG antibodies and CTX‐1/RANKL were assessed in the OVX+IM group. The generation of anti‐mRANKL IgG antibodies was negatively correlated with CTX‐1 (*R* = ‐0.6299, *P* = 0.0509, Figure 5B, left) and sRANKL (*R* = ‐0.687, *P* = 0.0282, Figure 5B, right) levels in mouse serum. However, we observed a positive linear correlation between BMD and anti‐RANKL IgG antibodies (R = 0.6461, *P* = 0.0027, Figure 5C).

To determine whether mRANKL‐MT3 immunization influences Th1 and Th2 cytokine production, we evaluated the effects of mRANKL‐MT3 on IL‐4 and IL‐10 secretion, which are markers for Th2 responses, and IFN‐γ, a marker for Th1 responses, in the culture supernatant of isolated spleen cells stimulated with sRANKL or mRANKL‐MT3 (Figure 5D). In Sham and IM‐stimulated splenocytes, there were no significant differences in IFN‐γ levels in the presence of sRANKL or mRANKL‐MT3. However, IL‐4 and IL‐10 secretion significantly increased due to sRANKL or mRANKL‐MT3 treatment in Sham and IM‐stimulated splenocytes, suggesting that anti‐RANKL production by mRANKL‐MT3 vaccination is Th2‐B cell‐mediated.

To examine antiserum effects on osteoclastogenesis, we treated primary BMMs with antiserum obtained from PBS‐ or mRANKL‐MT3‐immunized mice. While antiserum obtained from PBS‐immunized mice showed no effect on inhibition of osteoclastogenesis, the formation of TRAP‐positive multinucleated cells was significantly decreased by mRANKL‐MT3‐immunization‐induced antiserum, even at a dose of 1:4000 (Figure 5E). In addition, antiserum from mRANKL‐MT3‐immunized mice significantly inhibited bone resorption activity (Figure 5F). Notably, antiserum from mRANKL‐MT3‐immunized mice caused a slight decrease in NFATc1 and significantly downregulated TRAP, OSCAR, and other osteoclastogenic mRNA (Figure 5G; Figure S3a). In contrast, antiserum from PBS‐ and mRANKL‐MT3‐immunized mice did not decrease MAPK, AKT, or GSK‐3 levels (Figure S3b).

### 3.5 Effect of anti‐RANKL IgGs in sRANKL‐induced mice

In light of the above data, we further evaluated the effect of anti‐RANKL IgGs obtained from mRANKL‐MT3‐immunized mouse sera on bone metabolism and osteoclastogenesis. Anti‐RANKL IgGs were purified with a protein G column and confirmed by immunoblotting with sRANKL (Figure S4a,b).

**FIGURE 6:**
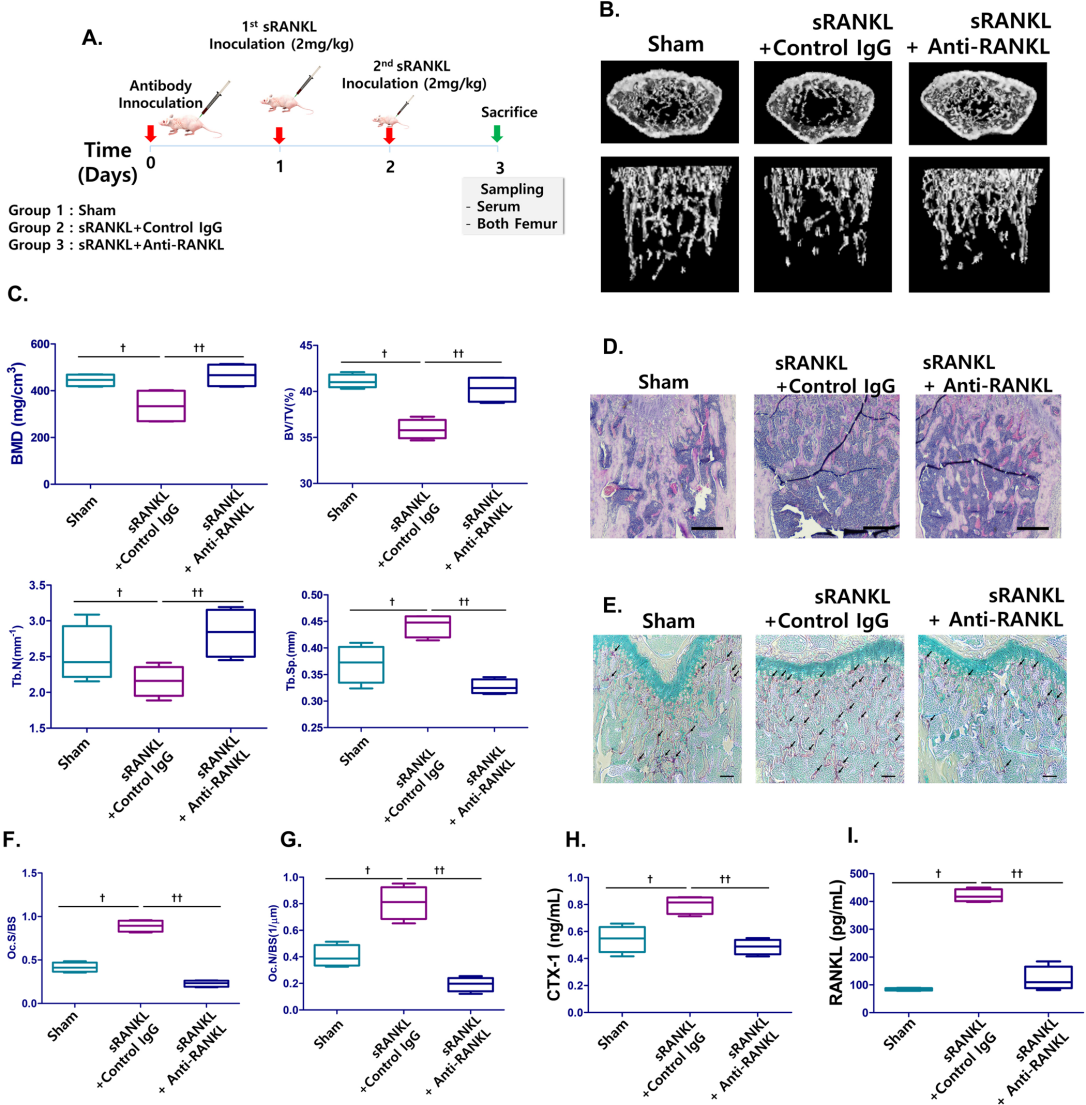
Effect of anti‐RANKL IgG induced by RANKL variants on sRANKL‐induced mice femurs. A, Immunization and sampling schedule in sRANKL‐induced mice. B, Three‐dimensional micro‐CT images revealed the trabecular bone architecture of the volume of interest in Sham‐, sRANKL with control IgG, and sRANKL with anti‐RANKL mice femurs (n = 10 images taken in total, one image for each mouse). C, Bone mineral density (BMD), bone volume/trabecular volume (BV/TV), trabecular number (Tb. N.), and trabecular separation (Tb. Sp.) are shown. D, Histomorphometric analysis. Magnification is 20×. The scale bar represents 500 μm. E, TRAP staining (n = 20 images taken in total, two images for each mousse) in the femurs. The arrow indicates TRAP positive cell. Magnification is 100×. The scale bar represents 50 μm. F, Parameters of femur osteoclasts. Oc.S/BS osteoclast surface per bone surface. G, Oc.N/BS, osteoclast number per bone surface. H, CTX‐1 and I, RANKL levels in mice sera. C, F, G, H, I, Error bars are mean ± SD. †*P* < .05 for Sham versus sRANKL + Control IgG group and ††*P* < .05 for sRANKL+Control IgG versus sRANKL+Anti‐RANKL

To investigate the effect of anti‐RANKL on osteolysis inhibition, purified anti‐RANKL from immunized mouse sera were inoculated in sRANKL‐treated mice (Figure 6A). As shown in the micro‐CT scan results, sRANKL‐treated mice showed fenestrated plate‐like structures of the trabecular bone and thinner rods forming the trabecular network, which finally degraded and left the structure less connected, indicating mild osteoporosis (Figure 6B). However, purified anti‐RANKL treatment markedly improved the trabecular bone architecture of VOI extracted from the distal femur in sRANKL‐induced mice, which was reduced in osteoporosis. Aside from visual assessment, BMD, BV/TV, Tb.N., Tb. Sp, and other bone scores were evaluated by quantitative micro‐CT (Figure 6C; Figure S4c). As expected, low BMD, BV/TV, and Tb.N. values in sRANKL‐induced mice were significantly rescued by anti‐RANKL treatment. These results clearly demonstrate the therapeutic effects of anti‐RANKL as an active immunogen for osteoporosis. Bone histomorphometric analysis and TRAP staining of mice femur were performed, revealing a fragmented network of the trabecular bone in sRANKL‐treated mice and an increased number of TRAP‐positive osteoclasts (Figure 6D and E; Figure S4d). However, a dense network with minimal spaces and fewer TRAP‐positive cells were observed in the presence of anti‐RANKL. These results confirmed that the OCs/BS% and OCs/mm^2^ values in sRANKL‐induced mice were significantly decreased after anti‐RANKL treatment (Figure 6F and G).

Also, circulating sRANKL and CTX‐1 levels were higher in sRANKL‐treated mice, while anti‐RANKL treatment significantly decreased circulating RANKL and CTX‐1 levels (Figure 6H and I). Additionally, OPG levels remained unchanged by sRANKL in either the presence or absence of anti‐RANKL (Figure S4e).

### 3.6 Effect of anti‐RANKL on sRANKL‐induced osteoclastogenesis *in vitro*

**FIGURE 7:**
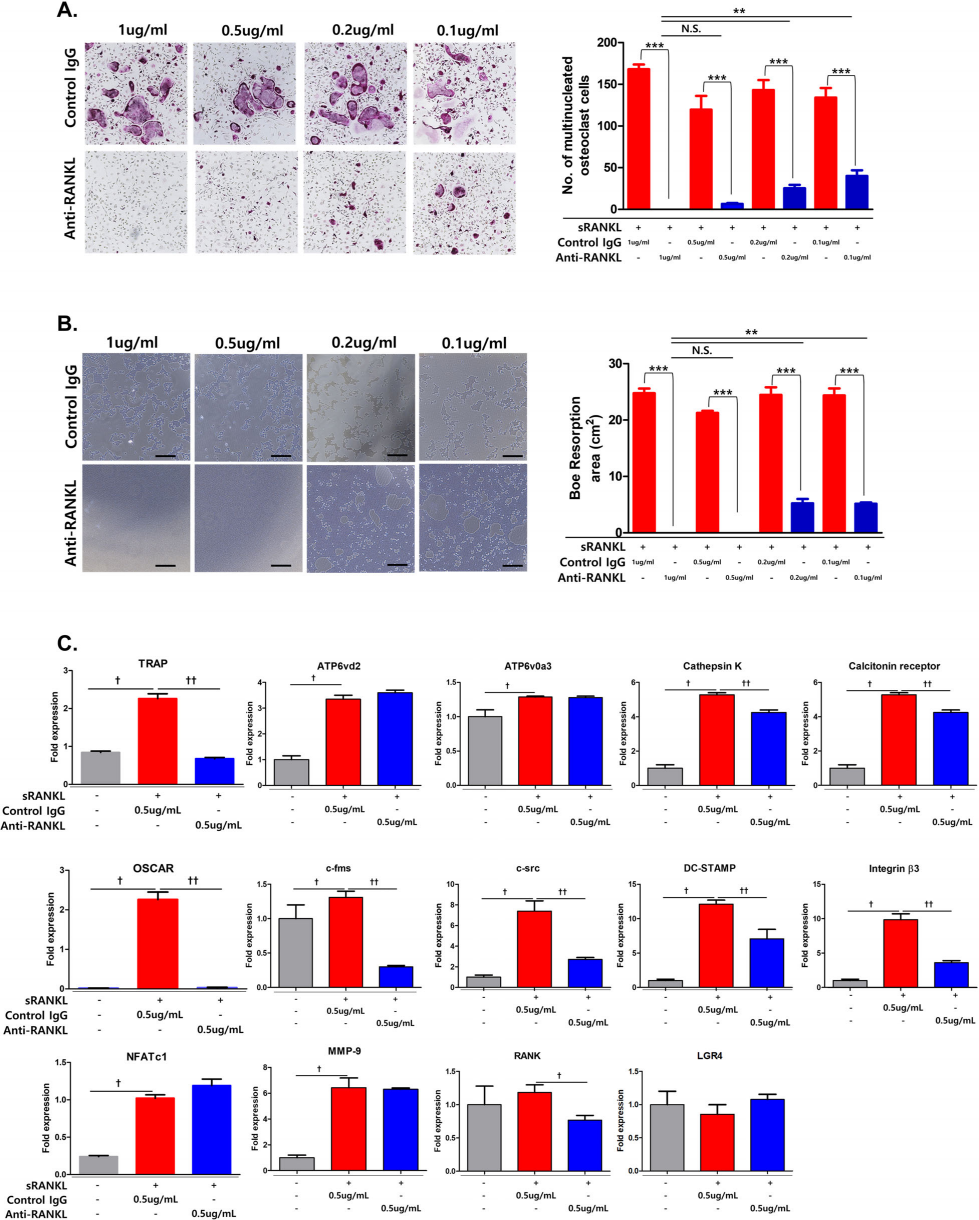
Effect of anti‐RANKL treatment on sRANKL‐induced osteoclastogenesis in BMMs. A, Effect of dose‐dependent anti‐RANKL immunization on TRAP‐positive cell generation compared to control IgG treatment. Representative TRAP staining images (upper panel) and TRAP‐positive multinucleated cell quantification (lower panel) in the presence of sRANKL (150 ng/mL). B, Effect of dose‐dependent anti‐RANKL immunization on bone resorption compared with control IgG treatment. All data are presented as the mean ± SD. N.S., not significant (*P* > .05), **P < .01 and ***P < .001. C, The mRNA expressions of TRAP, NFATc1, OSCAR, ATP6vd2, ATP6v0a3, calcitonin receptor, Cathepsin K, c‐fms, c‐src, DC‐STAMP, Integrin β3, MMP‐9, RANK, and LGR4 were analyzed by RT‐PCR of anti‐RANKL IgG (0.5 μg/mL) treated BMMs compared with control IgG treatment (0.5 μg/mL) in the presence of sRANKL. Error bars are mean ± SD. †*P* < .05 for Non. versus sRANKL + Control IgG group and ††*P* < .05 for sRANKL+Control IgG versus sRANKL+Anti‐RANKL

To investigate the effects of purified anti‐RANKL on the inhibition of osteoclastogenesis, primary BMMs were treated with purified anti‐RANKL from mRANKL‐MT3‐immunized mice in the presence of RANKL and M‐CSF. While commercial control IgG had no effect on osteoclastogenesis, TRAP‐positive multinucleated cells and bone resolving area were significantly reduced by anti‐RANKL‐treated BMMs, even at 0.1 μg/mL (Figure 7A and B). In addition, anti‐RANKL from mRANKL‐MT3‐immunized mice significantly decreased TRAP and OSCAR mRNA expression (Figure 7C). However, anti‐RANKL did not decrease NFATc1 levels.

**FIGURE 8:**
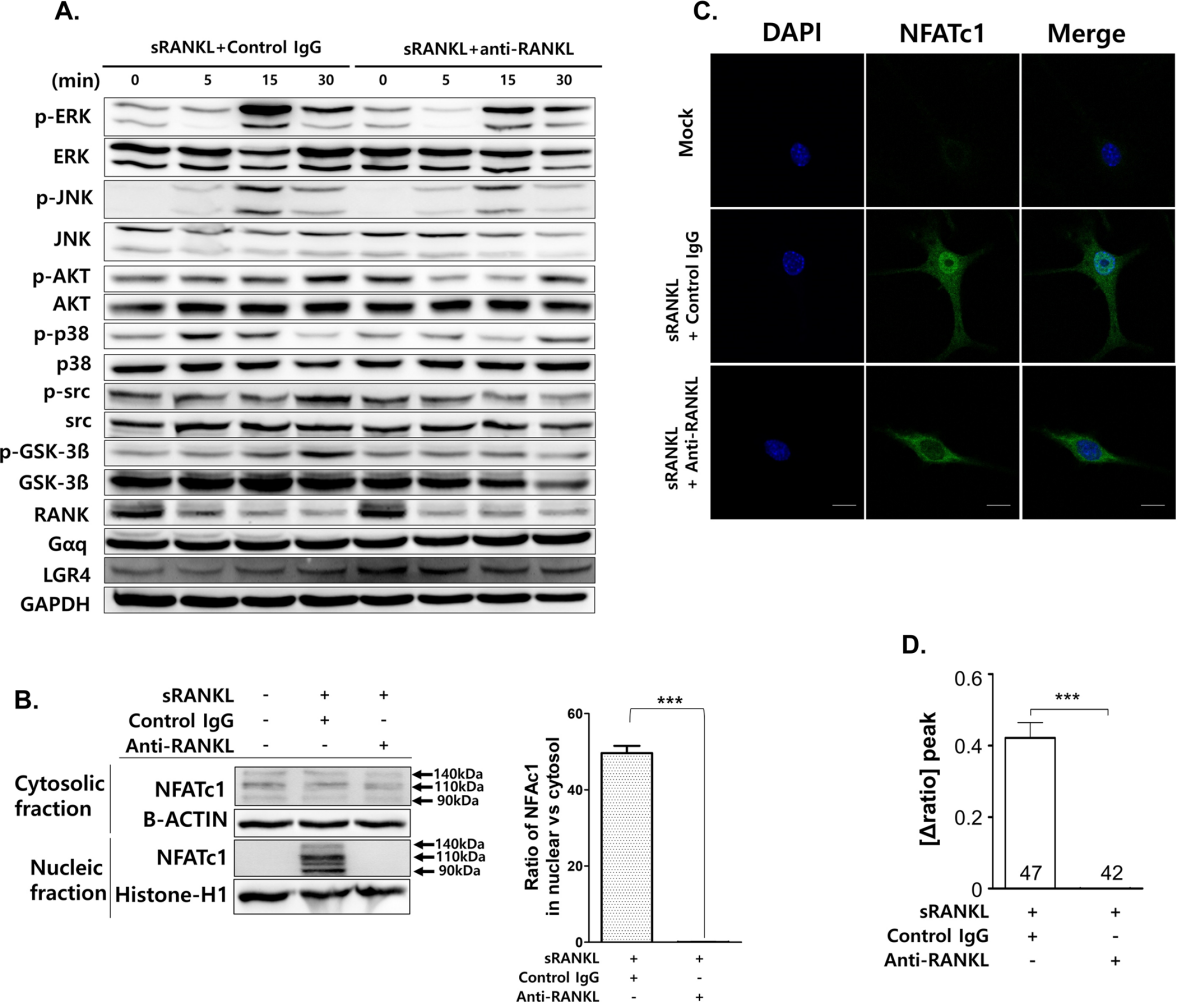
Effect of anti‐RANKL treatment on sRANKL‐induced signaling pathway in BMMs. A, Western Blot analysis. GAPDH was used as a loading control. Results are representative of three separate experiments with comparable results. B, NFATc1 nuclear translocation was analyzed by Western blot in cytosolic and nuclear fractions. Histone‐H1 from the nuclear fraction or β‐actin from the cytosol were used as loading controls. Densitometric analysis of NFATc1 in the cytosolic and nuclear fractions represents the mean ratio ± SD of three separate experiments. Significant differences were seen at ***P < .001 when comparing IgG with Anti‐RANKL IgG. C, Confocal microscopy images of NFATc1 nuclear translocation. Immunofluorescence images were acquired by staining for NFATc1 (green) and nuclei (blue). Magnification is 200×. The scale bar represents 20 μm. D, Intracellular calcium concentration ([Ca^2+^]_i_) was measured in BMMs. The numbers in *x*‐axis means counted BMM. Data were expressed as mean ± SD. ***P < .001

We verified that anti‐RANKL suppressed RANK and LGR4 signaling in a time‐dependent manner (Figure 8A). Treatment with sRANKL induced MAPK, AKT, src, and GSK‐3β phosphorylation. However, anti‐RANKL treatment significantly inhibited MAPK, AKT, src, and GSK‐3β phosphorylation. In addition, NFATc1 nuclear translocation was not detected by Western blot analysis or confocal microscopy in anti‐RANKL‐treated BMMs in the presence of sRANKL compared with control IgG (Figure 8B and C). Cytosolic calcium influx was also decreased by anti‐RANKL treatment in the presence of sRANKL (Figure 8D). Finally, we demonstrated that purified anti‐RANKL from mRANKL‐MT3‐immunized mouse sera blocked RANKL‐RANK and LGR4 signaling.

## 4 DISCUSSION

Osteoporosis has been considered a severe systemic bone disease characterized by a reduced bone mass, impairment in bone strength and microarchitecture, which lead to an increased possibility of osteoporotic fractures.^23^ Although current clinical treatments manage to improve the therapeutic possibilities of osteoporosis, such treatments are applied to the patients with established disease, and the question of prevention is not addressed.^24^ Therefore, the present study investigated whether preventive treatment is possible by immunization with RANKL, a key molecule of osteoclastogenesis. Considering that RANKL‐RANK signaling regulates the differentiation and activity of osteoclasts and the application of Denosumab (human anti‐RANKL mAb) in clinical therapies of osteoporosis reducing bone loss caused by bacterial infections, designing and estimating a novel vaccine targeting the RANKL is theoretically possible.^25^ Here, we developed a novel vaccine targeting RANKL by modifying five RANK‐binding amino acids in RANKL with conservative substitutions to similar but different amino acid residues. We hypothesized that modifying five RANK‐binding amino acids in RANKL with similar residue would not bind to RANK and generate the antibody of RANKL as a foreign body or stimulate the compensation signaling cascade against RANKL as a similar structure of RANKL.

As a vaccine on osteoporosis, mRANKL‐MT3 should have no increase in osteoclastogenesis and generate efficient anti‐RANKL antibodies in the living body. Chosen amino acid substitutions have been made with respect to domain structure with fewer hydrogen bonds and lower binding affinities. In the present study, mRANKL‐MT3 has the lowest osteoclastogenic activity among RANKL vaccine candidates in the presence of wild‐type RANKL with no increase in expression of osteoclastogenesis‐involved genes. On the other hand, mRANKL‐MT3 is considered to interact with other molecules such as LGR4, which is known as another RANKL receptor competing with RANK for RANKL binding in osteoclasts.^18^ LGR4 is considered to be a novel receptor of RANKL inhibiting RANKL‐induced osteoclast differentiation by blocking RANK–TRAF6 pathway, as well as through GSK‐3beta‐mediated inhibition of NFATC1.^18^ Furthermore, LGR4 is a compensative target within RANKL–RANK signaling, which suggests that LGR4 pathway in a negative feedback loop to limit RANKL osteoclastogenesis and its activities.^26^ In the present study, mRANKL‐MT3 treatment inhibited osteoclast differentiation in the presence of wild type RANKL *in vitro*, suggesting that this may be a direct active strategy for the treatment of bone‐resorbing diseases in clinics. Our data implicate LGR4 as a pivotal player of a negative‐feedback mechanism controlling osteoclast activities. LGR4 signaling cascades inhibit RANKL–NFATC1 signaling and cytosolic calcium release during osteoclast differentiation. Furthermore, the LGR4‐extra cellular domain is known to have a lower binding affinity than OPG with RANKL; thus, it is considered that LGR4‐mRANKL‐MT3 binding has little physiological effect in normal mice. This implies that the minimal effect of LGR4‐mRANKL‐MT3 protein complex in normal mice could be due to an endogenous OPG competition.^18^ In a previous study, *Lgr4* expression was reported to be increased during osteoclast differentiation peaking in mature osteoclasts; thus targeting LGR4 may affect mature osteoclasts but have less effect in pre‐osteoclasts and BMMs either, and thus reduce the side effects involved in the osteoclast‐related diseases mRANKL‐MT3 treatment.^18^

Here, we showed the effects of RANKL immunization on osteoclastogenesis *in vitro* and *in vivo*. Results revealed that mRANKL‐MT3 immunization generates a high‐titer antibody that efficiently recovered BMD (bone mineral density). Especially, mRANKL‐MT3 induced high serum IgG titers possibly due to residues F223 and Y224 which are close to the DE loop region of RANKL (residues 245–251), which can bind to Denosumab.^26^, ^27^ Denosumab, a human monoclonal antibody against RANKL, is a well‐known RANKL‐binding agent developed to treat osteoclast‐related diseases, including osteoporosis.^28^, ^29^ We also measured RANKL and OPG levels from the serum of control and immunized mice. As shown by the results, serum RANKL levels increased especially in the OVX group, providing a potential explanation for OVX‐induced bone erosion, which is mainly mediated by osteoclasts. However, serum RANKL levels decreased in the OVX+IM group negatively correlated with a high anti‐RANKL titer, due to neutralized serum RANK by mRANKL‐MT3 immunization, protecting OVX mice from bone erosion. The OVX‐induced osteoporotic mouse model is a recognized experimental animal model for the study of osteoporosis.^30^ Complex process induces the bone resorption, including the recruitment, adhesion, and differentiation of pre‐osteoclast cells before differentiation into mature osteoclasts. During this process, the OPG/RANKL/RANK pathway mediates the formation and development of osteoclast and plays a major role in regulating osteoclast activity.^5^, ^31^ As such, the vaccination of RANKL may be an alternative therapeutic approach for osteoporosis. The therapeutic vaccine also has been reported in previous studies, such as active immunization of OVX mice with RANKL covalently linked to human RANKL‐TNF‐like core fusion protein (RTFP‐2), virus‐like particles (VLP), Dickkopf‐1‐ derived peptides, and interspecies RANKL mutation.^15^, ^32^, ^33^, ^34^, ^35^, ^36^ However, vaccines introducing small particles or peptides may induce undesired immune responses. Interestingly in the present study, Th2 dependent cytokine production in spleen cells from immunized mice increased, although the level of inflammation did not alter significantly. This may be explained by the special role of RANKL in the immune system.

Over the last few decades, potential therapeutic strategies for targeting RANKL using by monoclonal antibodies and chemicals, have been developed.^25^, ^37^ Compared with antibodies and other biologics, vaccines may be a better choice for the treatment of chronic disease because of their distinct advantages, such as low‐costs and small protein doses required to induce its effects.^38^ Once an immune response in a host is triggered by vaccine, it is easily maintained by boosters.^39^ Traditional vaccines were constructed with conventional carriers that may have unanticipated side effects and complications.^40^ In a previous study, we constructed a RANKL vaccine, by substituting five residues within mRANKL. By the addition of histec, we successfully generated anti‐RANKL antibodies in OVX‐ or RANKL‐induced mice leading to a decrease in trabecular bone erosion without any undesired immune response.^16^ In the view of vaccination against osteoporosis, we further evaluated the efficacy of anti‐RANKL in the sRANKL‐induced osteoporosis model. We isolated and established antiserum titers for mice immunized with mRANKL‐MT3 and evaluated the ability of the isolated serum and IgG to regulate RANKL‐mediated osteoclast differentiation in vitro and in vivo. As expected, our results showed that the inhibition of osteoclastogenesis and bone erosion were induced not only by anti‐serum, but also by purified anti‐RANKL. Moreover, anti‐RANKL significantly decreased both signaling cascade RANK‐RANKL and LGR4‐RANKL in sRANKL‐induced osteoclastogenesis *in vitro* and *in vivo*. In the present study, mRANKL‐MT3 immunization had a dual effect in inhibiting RANKL activity by a comparative inhibition of the RANK‐RANKL signaling cascade and by generating anti‐RANKL. Thus, utilizing site‐specific incorporation of similar immunogenic amino acid residues into a protein may help avoid an undesired response. However, further studies are to needed to investigate the safety of the present vaccine. RANKL is expressed by T helper cells and also plays a role in DC (dendritic cell) maturation.^41^, ^42^ Therefore, additional researches are also required to determine how the effects of mRANKL‐MT3 immunization influence T helper cell subsets and DC phenotype in an osteoporosis mouse model.

The present study has several strengths and limitations. One potential advantage of a vaccine‐type approach to RANKL inhibition compared with antibody‐based approaches is that patients who discontinue denosumab experience rapid increases in bone remodeling and an increased risk of multiple vertebral fractures. It is possible, though clearly unproven, that this vaccine approach may not induce rapid high‐turnover bone loss, either by causing more durable RANKL inhibition or by allowing a more gradual resumption of remodeling when vaccinations are discontinued. It would be reasonable that this vaccine approach could be tested for its ability to minimize the risk of high‐turnover bone loss in situations where vaccinations and boosters are discontinued. One limitation of the present study is a lack of validation of safety or efficacy beyond long‐term rodent studies, despite their experimental reality approach. Because generated mutant RANKL is transformed from mouse RANKL, a small number of amino acid sequence is different from human RANKL and the clinical relevance of the present results for human study thus remains unclear. Therefore, it needs to be carried out in human RANKL knock‐in mouse model using by human RANKL mutant variant in further study. In addition, LGR4 signaling pathway is known to stimulate the osteoblast differentiation. Therefore, it needs to be carried out to validate the effect of mutant RANKL on osteoblast differentiation. Another limitation is the maintenance of osteoprotegerin (OPG) binding sequence in the mutant RANKL. The OPG acts as a naturally occurring decoy receptor for RANKL preventing it from binding to RANK expressed on osteoclast precursors or mature osteoclasts and is known that the monomeric cytokine‐binding region of OPG binds RANKL with ∼500‐fold higher affinity than RANK.^43^ Therefore, it needs to be carried out the trial to downregulate the binding affinity with OPG to evade the disruption by the OPG in further study.

## 5 CONCLUSION

In summary, our recombinant RANKL vaccine induces a dual inhibitory effect against RANKL during osteoclastogenesis. The first effect is due to RANKL‐LGR4 modulation of the RANKL–NFATc1 signaling cascade by a negative‐feedback mechanism, which controls osteoclast activity. The second effect is due to anti‐RANKL generation by mutant RANKL, which inhibits osteoclastogenesis and bone erosion. These results suggest that our point‐substitution RANKL mutant may pioneer the development of a novel vaccine to treat osteoporosis. Further research is required to resolve this question and to investigate LGR4 and anti‐RANKL modulation of RANKL signaling in other systems, including the immune system. Moreover, further studies are required to explore vaccine safety and compare the effects between our vaccine and monoclonal antibody therapy such as denosumab. Further experiments should address the use of this strategy in other models and in humans.

## CONFLICT OF INTEREST

The authors declare no potential conflict of interest.

## ETHICS APPROVAL AND CONSENT TOPARTICIPATE

All animal experiments were carried out in compliance with institutional and governmental requirements approved by the Institutional Animal Care and Use Committee (CIACUC2018‐S0012‐1) of Chosun University, Gwangju, South Korea.

## AUTHOR CONTRIBUTIONS

Y.K., H.S., Y.J. M.P., B.K., B.K., J.P., H.H., B.J., C.H., and W.L. designed and performed the research, analyzed, and interpreted data, and wrote the paper; Y.K., H.S., and Y.J. analyzed and interpreted the TRAP, FACS, RT‐PCR, and Western blot data and also contributed to some of the figures; W.L. performed and supervised RT‐PCR and Western blot data analysis; M.P. and B.K. provided ELISA assay assistance; Y.K., H.S., Y.J. M.P., and B.K. performed *in vitro* and *in vivo* studies under the supervision of H.H. and W.L.; B.K., performed intracellular calcium analysis under the supervision of C.H.; Y.K., H.S., and Y.J. performed histologic analysis under the supervision of W.L.; J.P. performed Micro‐CT analysis and contributed to some of the figures; H.S. performed pathological review of samples; Y.K. and Y.J. stained and scored the IHC data; W.L. analyzed publicly available datasets and supervised the study.

## Supporting information

Supplementary Figure 1. Effects of RANKL treatment variants on the osteoclastogenesis. (a) Actin filaments formation of rhodamine‐conjugated phalloidin‐stained cells were visualized under a fluorescence microscope (200× magnification). The scale bar indicates 100 μm. (b) The mRNA expression of ATP6vd2, ATP6v0a3, Cathepsin K, c‐fms, c‐src, calcitonin receptor, DC‐STAMP, Integrin β3 and RANK was analyzed by RT‐PCR. The data were normalized to GAPDH expression and are shown as the mean ratio ± SD from three separate experiments. Significant differences were depicted at *p < 0.05 when comparing RANKL‐WT with RANKL‐MT3.

Supplementary Figure 2. Comparative inhibition of osteoclastogenesis by RANKL variant. (a) Representative flow cytometry plots showing GST‐RANKL binding BMMs. Y axis represents GST binding BMMs. CD14 is used as a monocyte marker. (b) The mRNA expression of ATP6vd2, ATP6v0a3, calcitonin receptor, Cathepsin K, c‐fms, c‐src, DC‐STAMP, Integrin β3, MMP‐9, RANK and LGR4 were analyzed by RT‐PCR. The data were normalized to GAPDH expression and are shown as the mean ratio ± SD from three separate experiments. Significant differences were depicted at *p < 0.05, ***P < 0.001 when comparing mRANKL‐WT with mRANKLWT+mRANKL‐MT3. (c) NFATc1 nuclear translocation under confocal microscopy. Immunofluorescence images were acquired by staining for NFATc1 (green) and the nucleus (blue). Magnifications are 100Χ. Size bar is 50 μm.

Supplementary Figure 3. Effect of anti‐sera treatment on sRANKL‐induced osteoclastogenesis of BMMs. (a) mRANKL variants immunization antiserum titers and its effects on osteoclastogenesis. The mRNA expression levels of ATP6vd2, ATP6v0a3, calcitonin receptor, Cathepsin K, c‐fms, c‐src, DC‐STAMP, Integrin β3, MMP‐9, RANK and LGR4 were analyzed by RT‐PCR for each anti‐serum (1:1000) treated BMMs in the presence of sRANKL compared to ALD treatment. All data are presented as the mean ± SD of three measurements. ^†^P < 0.05 for Sham versus sRANKL + Control Serum group and ^††^P < 0.05 for sRANKL + Control Serum group versus sRANKL+Immunized Serum and * p < 0.05 for sRANKL + Control Serum group versus sRANKL+Sodium Alendronate (10μM) treated group. (b) Western Blot analysis. GAPDH was used as a loading control. Results are representative of three separate experiments with comparable results.

Supplementary Figure 4. Effect of anti‐RANKL treatment by RANKL variant on sRANKL‐induced mice femurs. (a) SDS phages from mock and immunized mice sera after purification using by protein G column. (b) Immunoblot of sRANKL and mtRANKL‐WT with mock mouse serum (left) or mRANKL‐MT3 immunized mouse serum (middle) or purified antibody as Anti‐RANKL. (c) Bone surface density (Bone surface/total volume), Cortical Bone area (Ct.Ar.), Cortical bone thickness (Ct.Th.) and Trabecular volume (TV) are shown; Error bars are mean ± S.D. †P < 0.05 for Sham versus sRANK+Control IgG, ††P < 0.05 for sRANK+Control IgG versus sRANK+Anti‐RANKL. (d) TRAP staining images of femurs. Magnifications are 20Χ. Size bar is 200 μm. (e) OPG level in the mice sera. Error bars are mean ± S.D. N.S.: non‐significant (P > 0.05).

Figure Legend

## Data Availability

All requests for raw and analyzed data and materials will be promptly reviewed to verify whether the request is subject to any intellectual property or confidentiality obligations by the corresponding author and Chosun University, Republic of Korea.
