# Supplementary material for: A novel modified RANKL variant can prevent osteoporosis by acting as a vaccine and an inhibitor
Source: Clin Transl Med. 2021 Mar 17;11(3):e368. doi: 10.1002/ctm2.368 (PMC7967917; doi:10.1002/ctm2.368)
Supplement: Supplementary file 5 — Figure Legend [file CTM2-11-e368-s004.docx]

**Supplementary Figure Legends**

Supplementary Fig.1. Effects of RANKL treatment variants on the osteoclastogenesis. (a) Actin filaments formation of rhodamine-conjugated phalloidin-stained cells were visualized under a fluorescence microscope (200× magnification). The scale bar indicates 100 μm. (b) The mRNA expression of ATP6vd2, ATP6v0a3, Cathepsin K, c-fms, c-src, calcitonin receptor, DC-STAMP, Integrin β3 and RANK was analyzed by RT-PCR. The data were normalized to GAPDH expression and are shown as the mean ratio ± SD from three separate experiments. Significant differences were depicted at *p < 0.05 when comparing RANKL-WT with RANKL-MT3.

Supplementary Fig.2. Comparative inhibition of osteoclastogenesis by RANKL variant. (a) Representative flow cytometry plots showing GST-RANKL binding BMMs. Y axis represents GST binding BMMs. CD14 is used as a monocyte marker. (b) The mRNA expression of ATP6vd2, ATP6v0a3, calcitonin receptor, Cathepsin K, c-fms, c-src, DC-STAMP, Integrin β3, MMP-9, RANK and LGR4 were analyzed by RT-PCR. The data were normalized to GAPDH expression and are shown as the mean ratio ± SD from three separate experiments. Significant differences were depicted at *p < 0.05, ***P<0.001 when comparing mRANKL-WT with mRANKLWT+mRANKL-MT3. (c) NFATc1 nuclear translocation under confocal microscopy. Immunofluorescence images were acquired by staining for NFATc1 (green) and the nucleus (blue). Magnifications are 100Χ. Size bar is 50 μm.

Supplementary Fig.3. Effect of anti-sera treatment on sRANKL-induced osteoclastogenesis of BMMs. (a) mRANKL variants immunization antiserum titers and its effects on osteoclastogenesis. The mRNA expression levels of ATP6vd2, ATP6v0a3, calcitonin receptor, Cathepsin K, c-fms, c-src, DC-STAMP, Integrin β3, MMP-9, RANK and LGR4 were analyzed by RT-PCR for each anti-serum (1:1000) treated BMMs in the presence of sRANKL compared to ALD treatment. All data are presented as the mean ± SD of three measurements. ^†^P < 0.05 for Sham versus sRANKL + Control Serum group and ^††^P < 0.05 for sRANKL + Control Serum group versus sRANKL+Immunized Serum and * p < 0.05 for sRANKL + Control Serum group versus sRANKL+Sodium Alendronate (10μM) treated group. (b) Western Blot analysis. GAPDH was used as a loading control. Results are representative of three separate experiments with comparable results.

Supplementary Fig.4. Effect of anti-RANKL treatment by RANKL variant on sRANKL-induced mice femurs. (a) SDS phages from mock and immunized mice sera after purification using by protein G column. (b) Immunoblot of sRANKL and mtRANKL-WT with mock mouse serum (left) or mRANKL-MT3 immunized mouse serum (middle) or purified antibody as Anti-RANKL. (c) Bone surface density (Bone surface/total volume), Cortical Bone area (Ct.Ar.), Cortical bone thickness (Ct.Th.) and Trabecular volume (TV) are shown; Error bars are mean ± S.D. †P < 0.05 for Sham versus sRANK+Control IgG, ††P < 0.05 for sRANK+Control IgG versus sRANK+Anti-RANKL. (d) TRAP staining images of femurs. Magnifications are 20Χ. Size bar is 200 μm. (e) OPG level in the mice sera. Error bars are mean ± S.D. N.S.: non-significant (P > 0.05).
